# Time-varying voriconazole clearance during extracorporeal membrane oxygenation

**DOI:** 10.1128/aac.00098-26

**Published:** 2026-04-30

**Authors:** Hakeem Yusuff, Jessica Gadsby, Graziella Isgro, Vasileios Zochios, David Jenkins, Sarah Cooke, Hussain Mulla

**Affiliations:** 1 Department of Anaesthesia and Critical Care, Extracorporeal Membrane Oxygenation Unit, University Hospitals of Leicester NHS Trust 4490, Leicester, United Kingdom; 2 Department of Respiratory Sciences, University of Leicester 574216, Leicester, United Kingdom; 3 National Institute of Heath Research (NIHR) Leicester Biomedical Research Centre, Glenfield Hospital 573772, Leicester, United Kingdom; 4 Department of Pharmacy, Glenfield Hospital, University Hospitals of Leicester NHS Trust 4490, Leicester, United Kingdom; 5 Department of Cardiovascular Sciences, University of Leicester 150459, Leicester, United Kingdom; 6 Department of Microbiology, University Hospitals of Leicester NHS Trust 4490, Leicester, United Kingdom; 7 College of Life Sciences, School of Healthcare, University of Leicester 98072, Leicester, United Kingdom; Providence Portland Medical Center, Portland, Oregon, USA

**Keywords:** pharmacokinetics, CYP2C19 genotype, therapeutic drug monitoring, time-varying clearance, drug sequestration

## Abstract

Invasive aspergillosis causes high mortality in critically ill patients, particularly those with viral pneumonitis receiving extracorporeal membrane oxygenation. Achieving effective voriconazole exposure in this setting is difficult, and existing data on drug concentrations during extracorporeal support are inconsistent. To characterize voriconazole pharmacokinetics in adults receiving extracorporeal membrane oxygenation and evaluate the influence of CYP2C19 genotype on drug exposure. This single-center prospective observational study included adults treated with intravenous voriconazole for suspected or confirmed aspergillosis during extracorporeal support. Serial plasma samples were analyzed using population pharmacokinetic modeling to describe drug disposition and simulate dosing regimens. Thirty-one patients (median age 40 years, mean weight 87 kilograms) provided 131 plasma samples; 61% carried reduced-function CYP2C19 variants. Voriconazole concentrations varied widely, with subtherapeutic levels increasing from 28% on days 1–5 to 47% by days 6–10. A dual-pathway model incorporating an early, rapidly decaying circuit sequestration process followed by a logistic rise in intrinsic clearance from 6.2 to 22.3 liters per h best described the data. Intermediate or poor metabolizers had 36% lower late-phase clearance, though genotype effects were estimated with substantial uncertainty. Simulations indicated that standard dosing achieved therapeutic concentrations in only half of patients at 48 h, declining sharply by day 7. Voriconazole clearance during extracorporeal membrane oxygenation is time-varying, with evidence of early circuit sequestration and later metabolic recovery influenced by CYP2C19 genotype. Early and repeated monitoring is required to maintain effective antifungal exposure. Time-varying clearance should inform dosing of voriconazole and other hepatically metabolized agents during extracorporeal support.

**CLINICAL TRIALS:**

This study is registered with ClinicalTrials.gov as NCT04868188.

## INTRODUCTION

Invasive aspergillosis contributes substantially to mortality in critically ill patients, extending beyond traditional immunocompromised populations to previously healthy patients with severe acute respiratory failure (1, 2). Viral pneumonitis has emerged as a particular risk factor for secondary fungal infections. Influenza-associated pulmonary aspergillosis occurs in up to 20% of patients with severe influenza requiring intensive care unit admission, with mortality approaching 50% even with treatment (3–6). Similarly, COVID-19 has been associated with high rates of secondary aspergillosis. Among patients receiving extracorporeal membrane oxygenation (ECMO) support for refractory respiratory failure, mortality rates of up to 78% have been reported in those with invasive aspergillosis (7, 8).

Voriconazole remains the first-line antifungal for invasive aspergillosis (9), yet achieving therapeutic plasma concentrations (2–5.5 mg/L) in critically ill patients is challenging, with both subtherapeutic exposure and toxicity frequently observed (10, 11). ECMO, now an established rescue therapy for severe respiratory failure (12, 13), further complicates voriconazole pharmacokinetics. Drug loss may occur through sequestration to the circuit’s tubing and oxygenator membranes; voriconazole’s moderate lipophilicity (log *P* ≈ 1.8) and protein binding (~58%) favor partitioning into hydrophobic circuit components, predisposing it to early underexposure (14–17). Concurrently, the physiological disturbances of critical illness—altered perfusion, systemic inflammation, corticosteroid use, and recovery of hepatic metabolism—can cause dynamic changes in drug distribution and clearance (11, 18).

Published reports on voriconazole exposure during ECMO are conflicting. Some describe low concentrations consistent with sequestration and increased clearance, whereas others report elevated levels suggestive of impaired metabolism early in critical illness (17, 19–24). These discrepancies leave clinicians without clear guidance on dosing in this high-risk population, a gap made more urgent by the rising incidence of influenza- and COVID-19-associated aspergillosis among ECMO-treated patients.

Voriconazole undergoes extensive hepatic metabolism, primarily via the polymorphic CYP2C19 enzyme, with additional contributions from CYP3A4 and CYP2C9. Genetic variants in CYP2C19 create a spectrum of metabolizer phenotypes. The *2 and *3 alleles are loss-of-function variants, and the *17 allele is a gain-of-function variant; patients are classified as normal (*1/*1), intermediate (*1/*2, *1/*3), poor (*2/*2, *2/*3, *3/*3), rapid (*1/*17), or ultrarapid (*17/*17) metabolizers (26), ranging from poor metabolizers who accumulate drug to ultrarapid metabolizers who may never achieve therapeutic levels (25, 26). Critical illness compounds this variability: inflammatory cytokines can suppress CYP activity, while corticosteroid exposure and recovery can accelerate metabolism (11, 27–30). As inflammation resolves, particularly with corticosteroid exposure common in ECMO patients, enzyme activity may rebound or become upregulated (31, 32).

Current voriconazole dosing guidelines make no adjustment for ECMO support, and standard therapeutic drug monitoring is typically delayed until day 3–5 (33), assuming stable pharmacokinetics—an assumption unlikely to hold given early sequestration and rapidly changing intrinsic clearance. Given that delayed antifungal therapy may increase mortality, understanding voriconazole exposure during ECMO is clinically urgent (34–36).

This study aimed to define voriconazole pharmacokinetics in critically ill patients receiving ECMO by evaluating CYP2C19 genotype impact and developing improved dosing recommendations. We utilized a prospective approach combining pharmacokinetic sampling, CYP2C19 genotyping, and population modeling to characterize drug disposition and identify optimal dosing strategies.

## MATERIALS AND METHODS

### Study design and population

This single-center, prospective, observational pharmacokinetic study was conducted at University Hospitals Leicester NHS Trust between August 2021 and May 2025. The study protocol was pre-registered on ClinicalTrials.gov (NCT04868188), and all procedures were conducted in accordance with STROBE (Strengthening the Reporting of Observational Studies in Epidemiology).

The target sample size (~30 participants, with up to five plasma samples per patient) was pragmatically determined by clinical incidence in a single tertiary ECMO center, with ~150 observations considered sufficient for robust population pharmacokinetic parameter estimation in nonlinear mixed-effects modeling. The influence of CYP2C19 genotype was a pre-specified but exploratory objective; prospective assurance of adequate representation across all metabolizer phenotypes was not feasible. Adults (≥18 years) receiving ECMO for acute severe respiratory and/or cardiac failure and initiated on intravenous voriconazole for suspected or confirmed invasive aspergillosis were eligible for inclusion. Pregnant patients, those with contraindications to voriconazole, and solid organ or hematopoietic stem cell transplant recipients were excluded. As an observational study embedded within routine clinical care, no randomization or treatment allocation occurred. All eligible patients were approached consecutively to minimize selection bias.

Voriconazole prescribing (initiation, dosing, and discontinuation) was determined independently by the treating clinicians in accordance with institutional protocols. Sampling procedures were standardized, bioanalysis was performed blinded to genotype and outcome, and potential confounders were prospectively recorded for covariate evaluation during model development.

### Voriconazole administration and sampling strategy

Voriconazole was infused intravenously over 1.5–2 h according to manufacturer guidance: 6 mg/kg loading dose (LD) every 12 h for the first two doses, then 4 mg/kg maintenance dose (MD) every 12 h. Dosing used adjusted body weight for obese (>20% ideal body weight) patients (37).

A pragmatic sampling design was adopted to balance data quality with clinical feasibility. Across three time windows (days 1–5, 6–10, and 11–14), up to five protocol-specified samples were collected per patient as follows: three samples in the initial window (1–3 h post-infusion for near-peak, 5–7 h for elimination, and pre-dose for trough), and single troughs during the intermediate and late windows. All samples were drawn exclusively from indwelling arterial lines, avoiding circuit ports, to ensure that measured concentrations reflect systemic drug exposure and are unaffected by oxygenator sequestration or recirculation artifact. The results were reviewed by the Chief Investigator as a safety precaution, but were not made accessible to the clinical team, except in the event of toxicity-related concerns.

Sampling was discontinued upon liberation from ECMO, cessation of intravenous voriconazole, or discharge from the ICU. Routine therapeutic drug monitoring samples requested by the clinical team during the study period were documented and incorporated into the analysis to enrich the data set while minimizing the need for additional blood sampling.

### Clinical data collection

Demographic data, ECMO parameters (mode, extracorporeal flow rate, circuit changes), and time-matched laboratory values were collected. All patients received extracorporeal support using the CentriMag system (St Jude Medical/Abbott) with Paragon oxygenators and circuit tubing (Chalice Medical, UK). Laboratory assessment included: hepatic function markers (ALT, bilirubin), renal function markers (serum creatinine), albumin, and inflammatory markers (C-reactive protein, procalcitonin). Concomitant medications, particularly corticosteroids and known CYP450 inhibitors or inducers, were recorded and included dose and duration of administration. All patients received proton pump inhibitor therapy (intravenous omeprazole or enteral lansoprazole); given universal use, PPI was not explored as a covariate in the pharmacokinetic model. Additional parameters included use of continuous renal replacement therapy, mean arterial pressure, and cumulative fluid balance.

### Laboratory analysis

Plasma voriconazole concentrations were measured using validated liquid chromatography-tandem mass spectrometry (Chromsystems, Germany). The assay was linear over the range 0.01–13.6 mg/L with a lower limit of quantification of 0.01 mg/L. Between-batch precision (coefficient of variation) across low, medium, and high QC levels averaged 5.5%, satisfying bioanalytical validation criteria. No voriconazole concentrations fell below the lower limit of quantification (0.01 mg/L); all measured concentrations were included in the population pharmacokinetic analysis.

CYP2C19 genotyping employed point-of-care PCR technology (Genomadix Cube System, Sela Medical, London, UK) using buccal swabs.

### Population pharmacokinetic analysis

Population pharmacokinetic modeling was conducted using Monolix 2024 (Lixoft, France) with the Stochastic Approximation Expectation-Maximization algorithm. Monte Carlo simulations were performed in Simulx (Monolix Suite), with post-processing and graphics generated in R (The R Foundation for Statistical Computing, 2023 version 4.3.1).

Initial structural model development evaluated one- and two-compartment models with first-order elimination. Given voriconazole’s known nonlinear pharmacokinetics, models with Michaelis-Menten elimination were also explored. Because critically ill patients supported with ECMO may experience both circuit-related drug losses and evolving intrinsic metabolic function, models with time-varying pharmacokinetic processes were additionally examined. These included alternative functional forms for clearance over time (logistic, exponential, and linear trajectories) and parallel pathways in which an apparent clearance term represented sequestration within the ECMO circuit and decayed mono-exponentially from an initial value.

Model selection considered objective function values (−2 log-likelihood), AIC, BIC, and parameter precision (relative standard error (RSE) <50% was considered acceptable). Interindividual variability was modeled using log-normal distributions. Residual variability was explored via proportional, additive, and combined error models. Missing concentration data were not imputed; analyses used all available observations.

Covariate analysis involved a structured stepwise approach. Covariates were first screened graphically and by plausibility. Continuous variables were centered on medians and evaluated as linear or power functions; categorical covariates were modeled as proportional shifts. Significance thresholds were *P* < 0.05 (ΔOFV ≥ 3.84) for forward inclusion and *P* < 0.01 (ΔOFV ≥ 6.63) for backward elimination. Missing covariate values were excluded from specific covariate analyses while retaining all concentration data in the population model. For serial laboratory values, the most recent prior measurement was carried forward when current values were unavailable.

### Model validation

Model evaluation included diagnostic plots (individual weighted residuals, normalized prediction distribution errors) and visual predictive checks (1,000 simulation). Parameter uncertainty was assessed using the Fisher information matrix. Monte Carlo simulations (5,000 virtual patients) evaluated probability of achieving therapeutic targets (2–5.5 mg/L) under standard and alternative dosing regimens.

## RESULTS

### Patient characteristics

Thirty-three adults receiving ECMO were enrolled. One patient was withdrawn following initiation of carbamazepine, a contraindicated interacting drug, and in one patient, voriconazole samples could not be collected within the defined sampling windows. The final pharmacokinetic analysis, therefore, included 31 patients. Median age was 40 years (range 19–58), with 17 females (55%) and mean weight 87 kg (range 49–134). Twenty-nine patients (94%) received venovenous ECMO; two (6%) received venoarterial ECMO. Primary ECMO indications were influenza-associated respiratory failure (*n* = 20, 65%), COVID-19 pneumonitis (*n* = 6, 19%), other severe respiratory conditions (*n* = 5, 16%), including near-fatal asthma and post-cardiac surgery complications. Twenty-seven patients (87.2%) received corticosteroids during voriconazole therapy. Median duration of corticosteroid use during the study period was 8 days (IQR 5–12), typically initiated within 24 h of ECMO commencement. The cohort was ethnically diverse (Table 1). Laboratory parameters are summarized in Table S1. One patient underwent a circuit change on day 13 of voriconazole therapy. The majority of patients (25/31) commenced voriconazole within 24 h of ECMO cannulation; the remaining initiated treatment on day 2 (*n* = 4), day 3 (*n* = 1), day 4 (*n* = 1), and day 7 (*n* = 1).

**TABLE 1:** Baseline characteristics of the study population (*n* = 31)^*a*^

Characteristic	Value
Age, years (mean [range])	39.9 [19–58]
Weight, kg (mean [range])	86.8 [49–134]
Female sex, *n* (%)	17 (54.8)
Male sex, *n* (%)	14 (45.2)
Ethnicity, *n* (%)	
White British	16 (51.6)
Asian	8 (25.8)
Mixed British	5 (16.1)
Other	2 (6.5)
ECMO indication, *n* (%)	
Influenza	20 (64.5)
COVID-19	6 (19.4)
Other	5 (16.1)
ECMO mode, *n* (%)	
Venovenous (VV)	29 (93.5)
Veno-arterial (VA)	2 (6.5)
ECMO duration, *n* (%)	
0-5 days	10 (32.3)
6-10 days	8 (25.8)
11-14 days	4 (12.9)
> 14 days	9 (29)
Concomitant CVVHDF, *n* (%)	
Full study duration	12 (38.7)
No CVVHDF	12 (38.7)
Intermittent/on–off	7 (22.6)
Concomitant corticosteroid (dexamethasone / prednisolone / hydrocortisone / methylprednisolone), *n* (%)	27 (87.2)

^*a*^ Mean (SD or range); CVVH, Continuous venovenous hemofiltration.

CYP2C19 genotyping revealed: 12 normal metabolizers (39%; *1/*1), 12 intermediate metabolizers (39%; *1/*2, *2/*17), five rapid metabolizers (16%; *1/*17), one poor metabolizer (3%; *2/*2), and one ultrarapid metabolizer (3%; *17/*17). Overall, 61% of patients carried variant alleles affecting voriconazole metabolism.

### Pharmacokinetic sampling and observed concentrations

A total of 131 voriconazole plasma samples were included in the modeling data set (median four per patient; range 1–7). Sampling variability reflected differing treatment durations: 22 patients had abbreviated sampling due to ECMO decannulation (*n* = 17) or intravenous voriconazole discontinuation (*n* = 5). Median time to first sample was 49.8 h post-initiation (range 2.6–111.2 h), capturing early drug exposure. Voriconazole concentrations varied widely, from 0.1 mg/L to 8.0 mg/L.

Trough concentrations declined progressively over time. Early-phase (days 1–5) troughs averaged 3.1 mg/L (range 0.1–7.1), dropping to 2.5 mg/L (range 0.2–5.1) in the intermediate phase and 2.1 mg/L (range 1–3.8) in the late phase. The proportion of subtherapeutic troughs (<2 mg/L) increased from 28% in days 1–5 to 47% by days 6–10.

### Population pharmacokinetic model development

Initial one- and two-compartment models with time-invariant clearance showed systematic bias, consistently underpredicting early voriconazole concentrations and overpredicting later concentrations. This pattern suggested that more than one time-dependent process was influencing drug disposition. Among the candidate models tested, those incorporating both a logistic increase in intrinsic clearance and a parallel sequestration pathway provided the best description of the data. The sequestration pathway was parameterized as an apparent clearance term (CLseq) operating in parallel with intrinsic clearance, with a high initial value that decayed mono-exponentially with half-life T½seq. This structure improved early predictions, capturing the rapid loss of drug during the first 24–48 h on ECMO, while the logistic intrinsic clearance term described the later increase in metabolic capacity during recovery.


$$CL_{intr}(t)=CL_{1}+\frac{CL_{2}-CL_{1}}{1+exp[-k(t-T_{switch})]}$$

$$CL_{seq}(t)=CL_{seq0}\cdot exp\left(-\frac{\ln2}{T_{1/2,seq}}t\right)$$

$$CL_{total}(t)=CL_{intr}(t)+CL_{seq}(t)$$

Where CL_intr_(t) is the time-varying intrinsic clearance, CL_seq_(t) is the exponentially decaying sequestration clearance, and CL_total_(t) is the sum of both processes. CL₁ and CL₂ are the initial and late intrinsic clearance values, T_switch_ is the logistic midpoint of the transition, k is the slope parameter (fixed at 0.1), CL_seq0_ is the initial sequestration clearance, and T_½,seq_ is the half-life of sequestration decay.

### Final model parameters and covariate effects

The final model estimated a central volume of distribution (V) of 144.8 L (RSE 8.0%). Intrinsic clearance increased from 6.22 L/h (CL1, RSE 24.8%) to 22.3 L/h (CL2, RSE 24.4%), with a transition midpoint at 88 h (RSE 29.4%). CYP2C19 genotype was retained as a covariate on late clearance based on mechanistic plausibility and model improvement during forward selection: patients carrying reduced-function alleles (intermediate/poor metabolizers) had 36% lower CL2 compared with normal metabolizers (β = −0.44, RSE 60%), though this estimate had substantial uncertainty. The sequestration pathway was characterized by an initial CL_seq0_ of 17.7 L/h (RSE 117%) and a T_½,seq_ of 4.2 h (RSE 84%). Although these parameters were estimated with substantial uncertainty, the inclusion of this pathway improved model predictions during the first 24–48 h, consistent with substantial early drug loss that decays rapidly as the circuit saturates. Interindividual variability (CV%) was moderate for V (28%), high for CL_1_ (77%), CL_2_ (87%), and timing of transition (82%) (Table 2). CYP2C19 genotype explained 10% of interindividual variability in steady-state clearance, with substantial residual variability remaining. Other potential covariates—inflammatory markers (CRP, procalcitonin), concomitant medications, ECMO mode, renal replacement therapy, hepatic function, and albumin—failed to meet retention criteria and provide meaningful reduction in unexplained variability. Although certain covariates showed suggestive associations (CRP with reduced clearance, ECMO flow with enhanced late clearance), their inclusion provided insufficient model improvement to justify increased complexity.

**TABLE 2:** Final population pharmacokinetic parameter estimates for voriconazole in adults on ECMO (updated)^*a*^^,^^*b*^^,^^*c*^^,^^*d*^^,*e*^

Parameter	Estimate	RSE (%)	95% CI	IIV (%CV)	Shrinkage (%)
V (L)	144.79	8.04	123.78–169.37	27.6	22.0
CL₁ (L/h)	6.22	24.8	3.90–9.92	77.0	14.1
CL₂ (L/h)	22.26	24.4	14.08–35.20	86.7	15.9
T_switch_ (h)	87.96	29.4	51.03–151.61	81.9	39.6
CL_seq0_ (L/h)	17.68	117.3	4.01–78.06	NC	NC
T½,seq (h)	4.17	83.7	1.23–14.14	NC	NC
Genotype effect on CL₂ (natural log-scale)	−0.44	60.0	−0.96–0.078	NC	NC
Residual additive error SD (mg/L)	0.091	61.3	0.034–0.25	N/[Table-fn T2_FN5]A	N/A
Residual proportional error	0.12	21.5	0.077–0.17	N/A	N/A

^*a*^ CL₁ and CL₂ represent intrinsic voriconazole clearance in the early and late phases of ECMO, respectively. T_switch_ is the midpoint of the clearance transition. CL_seq₀_ is the initial sequestration clearance into the ECMO circuit at t = 0 and T_½seq_ is the half-life (decay) of sequestration clearance. V is the central volume of distribution. Estimates are population means with relative standard error (RSE) and approximate 95% confidence intervals (Wald).

^*b*^ NC, not calculated.

^*c*^ Interindividual variability (IIV) shown as %CV from log-normal ω (CV = sqrt[exp(ω²) − 1] × 100).

^*d*^ Interindividual variability (IIV) shown as %CV from log-normal ω (CV = sqrt[exp(ω²) − 1] × 100).

^*e*^ N/A, not applicable.

### Model validation and diagnostic performance

**Fig 1:**
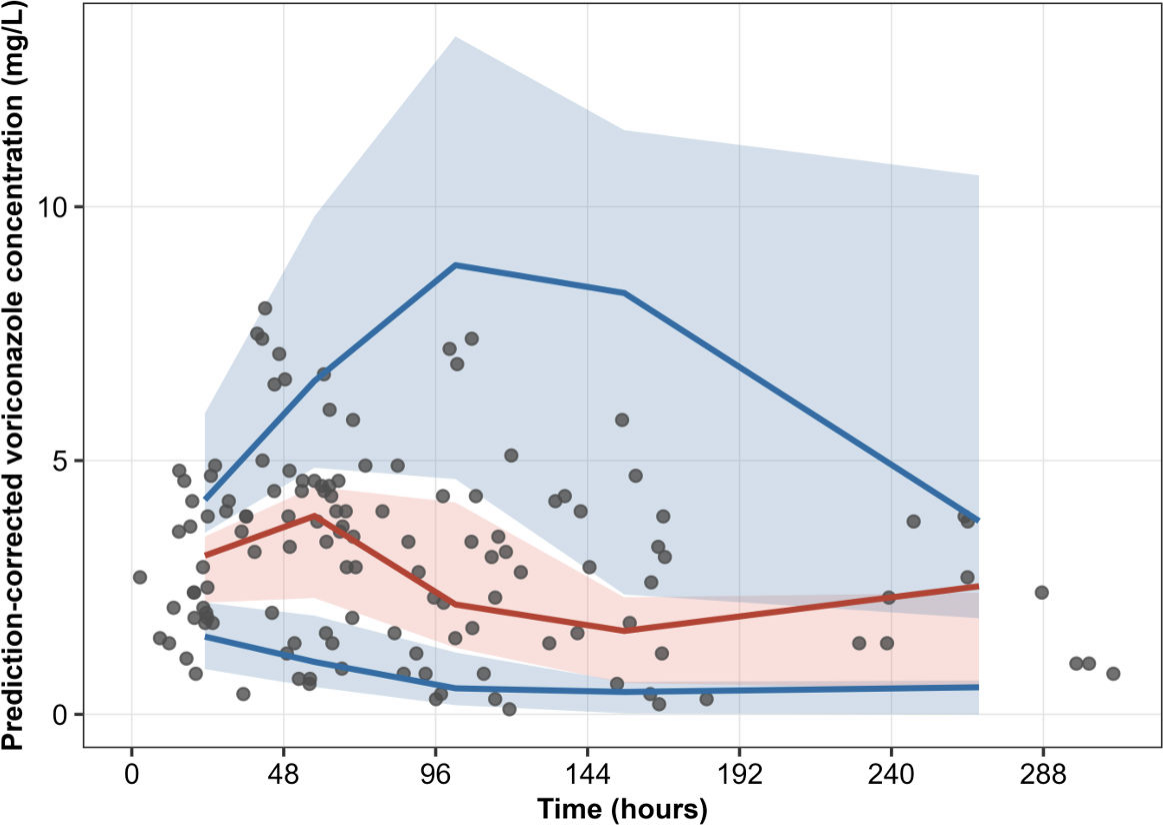
Prediction-corrected visual predictive check (VPC) of voriconazole concentrations in adults on ECMO. Observed concentrations are shown as points; empirical 5th, 50th (median), and 95th percentiles of the observations are shown as solid lines. Shaded areas represent 90% prediction intervals of the corresponding final model simulated percentiles.

Model validation demonstrated good overall fit and predictive performance. Observed–predicted and individual fit plots showed no systematic bias, and residuals were symmetrically distributed without time-dependent trends (Fig. S1 to S4). Prediction-corrected VPCs reproduced the early rise then decline and later variability in concentrations, with approximately 95% of observations within the nominal 90% prediction interval (Fig. 1). Compared with clearance-only models, the final dual-pathway model substantially reduced early bias, especially in the first 24–48 h, where sequestration accounted for drug losses not explained by metabolic clearance alone.

### Dosing simulations and target attainment

**Fig 2:**
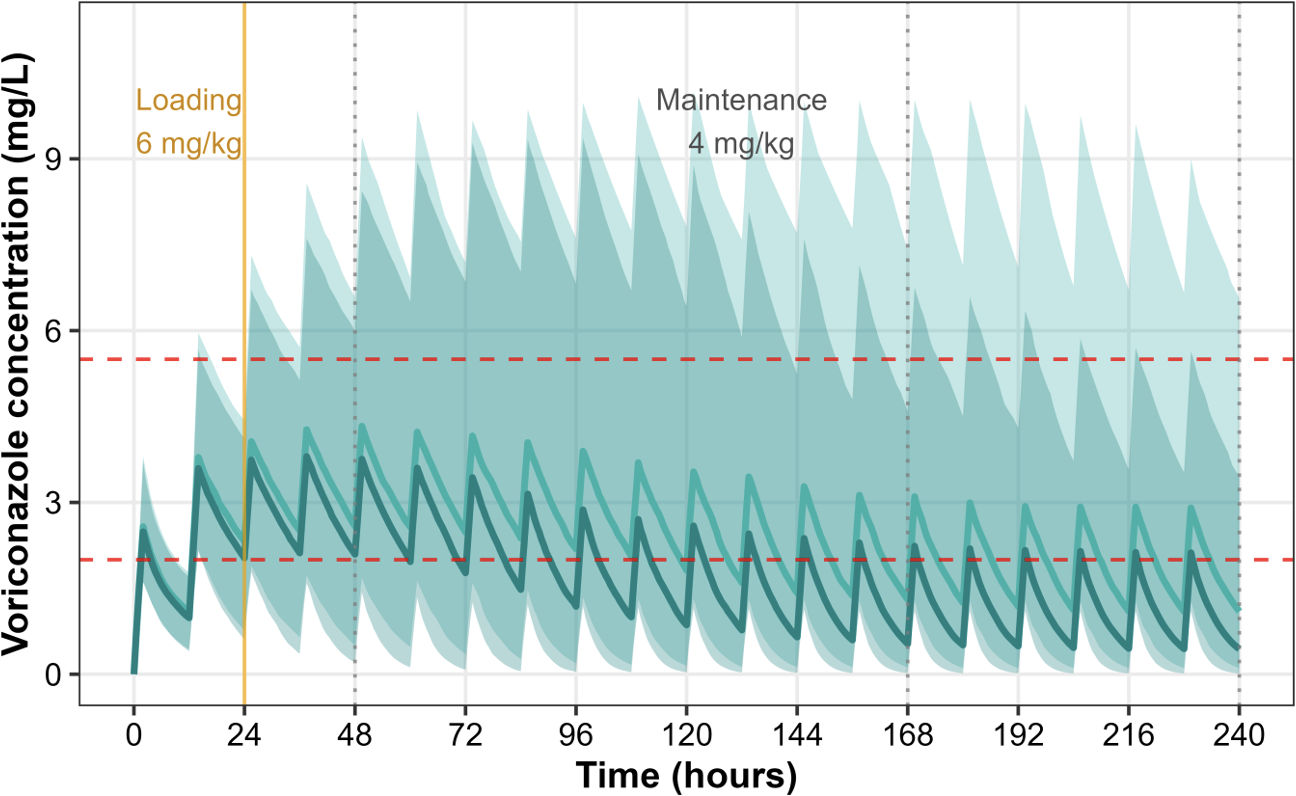
Monte Carlo simulation (*n* = 1,000) of voriconazole concentration–time profiles during ECMO stratified by CYP2C19 genotype. Simulated plasma concentrations (median and 90% prediction interval) are shown following standard SmPC recommended dosing: intravenous loading doses of 6 mg/kg every 12 h for two doses, then maintenance dosing of 4 mg/kg every 12 h. Dark teal shading represents wild-type (WT, normal/rapid metabolizers), and light teal shading represents intermediate/poor metabolizers (IM/PM). Horizontal dashed red lines indicate the commonly accepted therapeutic range (2-5.5 mg/L).

**Fig 3:**
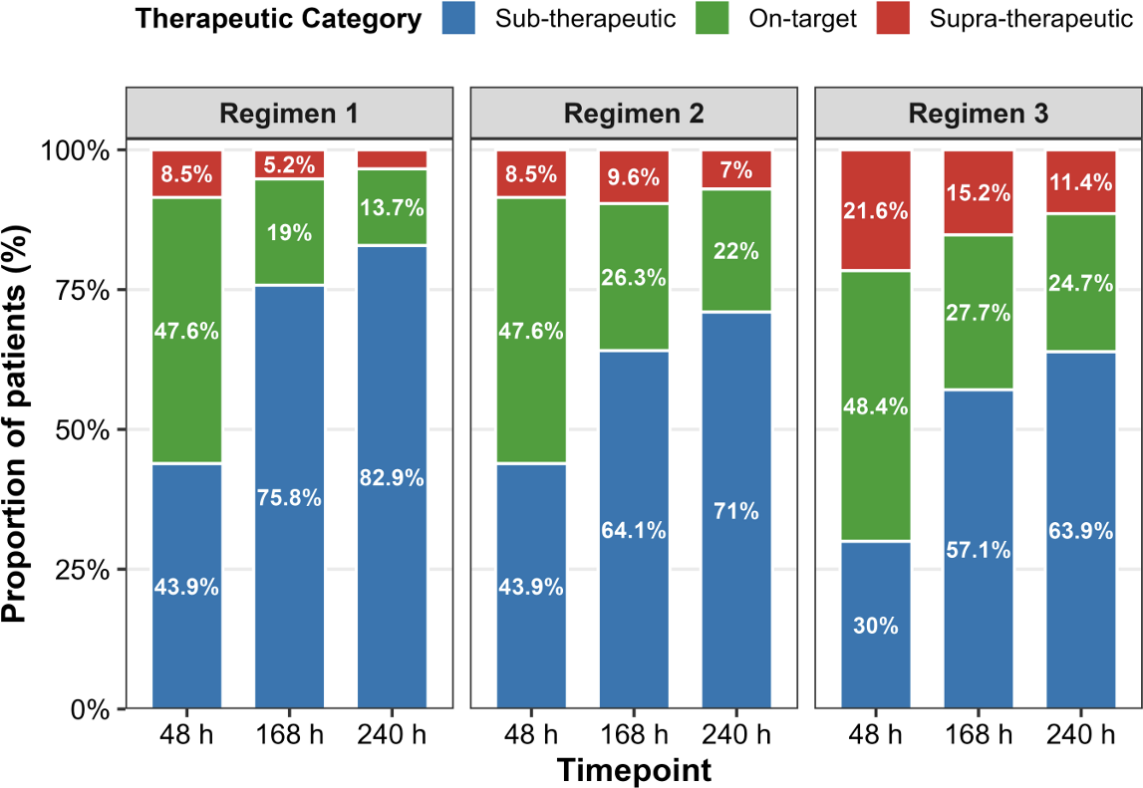
Probability of target attainment (PTA) for alternative voriconazole dosing regimens during ECMO. Stacked bar plots show the proportion of simulated patients (N = 1,000) within the therapeutic window (blue, 2–5.5 mg/L), subtherapeutic (<2 mg/L, green), or supratherapeutic (>5.5 mg/L, red) at 48 h, 168 h, and 240 h of therapy. Regimen 1 (SmPC dose): loading dose (LD) 6 mg/kg every 12 h for two doses, then maintenance dose (MD) 4 mg/kg every 12 h. Regimen 2: LD 6 mg/kg every 12 h for two doses, then MD 4 mg/kg every 12 h for eight doses, followed by MD 6 mg/kg every 12 h. Regimen 3: LD 8 mg/kg every 12 h for two doses, then MD 6 mg/kg every 12 h for eight doses, followed by MD 8 mg/kg every 12 h.

Monte Carlo simulations evaluated three intravenous regimens: (i) LD 6 mg/kg every 12 h for two doses followed by MD 4 mg/kg every 12 h (standard dosing according to SmPC) (Fig. 2) ; (ii) LD 6 mg/kg every 12 h for two doses, MD 4 mg/kg every 12 h for eight doses, then MD 6 mg/kg every 12 h; and (iii) LD 8 mg/kg every 12 h for two doses, MD 6 mg/kg every 12 h for eight doses, then MD 8 mg/kg every 12 h. Across all regimens, the proportion of patients within the therapeutic range was greatest at 48 h but declined sharply thereafter, with most patients subtherapeutic by day 7. The escalated dose regimens provided modest improvements in late exposure compared with standard dosing, but neither prevented the overall trend toward underexposure (Fig. 3).

## DISCUSSION

This prospective population pharmacokinetic study demonstrates that voriconazole clearance in patients receiving ECMO support is governed by two sequential processes: rapid early sequestration within the extracorporeal circuit, followed by a nearly fourfold rise in intrinsic clearance occurring predominantly over the first 3–5 days. These findings help reconcile apparently contradictory reports in the literature. Early-phase studies have described drug loss to the circuit and suppressed clearance, often associated with elevated plasma concentrations, whereas later-phase studies have reported increased clearance and predominantly subtherapeutic levels (17, 19–23). Other investigations, however, have found little or no effect of ECMO on voriconazole pharmacokinetics, likely reflecting sampling at intermediate or variable time points along this continuum (24, 38, 39). Rather than representing conflicting results, these studies collectively describe different phases of a dynamic process. Explicit modeling that incorporates both sequestration and time-varying clearance explains why standard dosing regimens frequently fail and provides a mechanistic framework for therapeutic optimization.

### Mechanistic considerations

**Fig 4:**
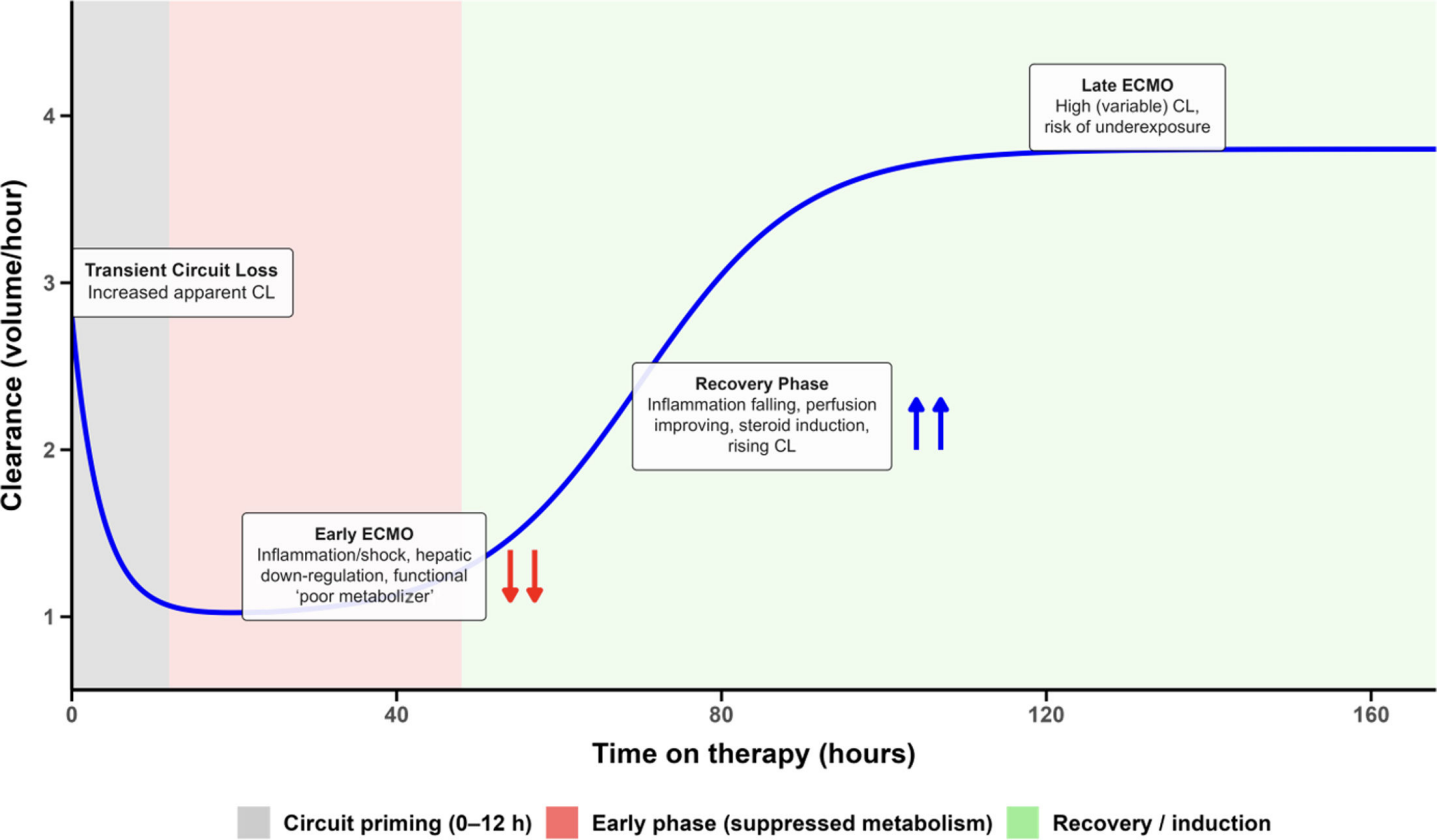
Conceptual model of dynamic drug clearance during ECMO. The schematic illustrates hypothesized phases of clearance for lipophilic, hepatically metabolized drugs during ECMO. An initial transient increase in apparent clearance may occur due to circuit sequestration (gray zone), followed by a period of suppressed clearance driven by inflammation, shock, and hepatic downregulation, mimicking a “poor metabolizer” state (red zone). As patients recover, clearance rises (green zone) as inflammation subsides, perfusion improves, and corticosteroid induction emerges. In later ECMO, clearance may be high but variable, creating a risk of underexposure. Downward arrows indicate phases where drug accumulation and toxicity risk predominate, while upward arrows indicate increased risk of subtherapeutic exposure as clearance recovers.

The biphasic clearance pattern reflects the evolving pathophysiology of ECMO-supported critical illness (Fig. 4). Initially, multiple mechanisms affect drug disposition. Circuit-related sequestration causes immediate drug loss through adsorption to tubing and oxygenator membranes—an effect most pronounced at ECMO initiation that diminishes as surfaces saturate. Simultaneously, severe systemic inflammation suppresses hepatic metabolism through cytokine-mediated downregulation of cytochrome P450 enzymes, functionally converting normal metabolizers into poor metabolizers (phenoconversion) (40). Studies demonstrate that C-reactive protein levels exceeding 96 mg/L correlate with markedly increased risk of supratherapeutic voriconazole concentrations (11, 18). Concurrent hemodynamic compromise—e.g., cardiogenic shock and non-pulsatile ECMO blood flow—further reduces hepatic perfusion and oxygen delivery (41). Together, these overlapping processes contribute to substantial early variability in voriconazole exposure. Although Michaelis-Menten elimination represents a plausible alternative explanation for the apparent rise in clearance over time, it was rejected during model development for two reasons. First, observed trough concentrations were not sufficiently elevated to support meaningful enzyme saturation: mean troughs declined progressively from 3.1 mg/L in days 1–5 to 2.1 mg/L in days 11–14, below the Km range typically reported for CYP2C19-mediated voriconazole metabolism. Second, the Michaelis-Menten model showed systematic underprediction of observed concentrations at 24–48 h, reflecting its inability to accommodate a genuinely suppressed early clearance state that transitions with time rather than with concentration.

As patients stabilize and recover, metabolic capacity not only returns but may exceed baseline function. Inflammatory resolution is associated with normalization of cytochrome P450 expression, while improving hemodynamics may contribute to restored hepatic perfusion. Corticosteroid exposure (observed in 87% of patients) may accelerate metabolic recovery through direct enzyme induction. Both dexamethasone and methylprednisolone are recognized inducers of CYP450, with previous reports documenting dramatic reductions in voriconazole concentrations following high-dose steroid administration (30–32). Notably, as all patients received concomitant PPI therapy—potent competitive CYP2C19 inhibitors—the late clearance rise is unlikely to reflect CYP2C19 recovery *per se* and more plausibly represents CYP3A4 and CYP2C9 induction and recovery alongside improving hepatic perfusion.

The observed late-phase clearance of 22 L/h substantially exceeds typical adult values of 3–11 L/h, suggesting convergence of multiple clearance-enhancing factors during recovery. This high value warrants scrutiny, as the model’s time-invariant V assumption attributes all concentration changes to clearance variations. Volume contraction with resolution of systemic inflammation during recovery could theoretically cause the model to overestimate clearance changes. However, sensitivity analyses fixing V at 100 L (versus 145 L estimated) only modestly reduced clearance estimates to 19 L/h, and models incorporating time-varying V showed no improvement in fit, supporting genuine clearance enhancement rather than modeling artifact. The gradual transition over approximately 88 h, with considerable interindividual variability, reflects heterogeneous recovery trajectories and varying exposure to modulating factors.

### Pharmacogenomic implications

Despite the profound physiological perturbations of critical illness, CYP2C19 genotype showed a clinically relevant association with voriconazole clearance throughout ECMO support. Poor and intermediate metabolizers – 42% of patients – demonstrated approximately 30% lower clearance in the late phase, suggesting that genetic factors retain clinical relevance even amid dynamic physiology. However, universal concomitant PPI use—competitive CYP2C19 inhibitors—likely attenuated genotype-related differences in clearance, and the true pharmacogenomic effect may be larger than observed in this cohort (40).

### Implications for clinical practice

These findings question current voriconazole dosing approaches in patients receiving ECMO. Standard practice delays therapeutic drug monitoring until day 5–7, yet simulations (Fig. 3) show that although initial target attainment is ~50% across regimens, most patients are already subtherapeutic by day 7. Neither intensified strategy overcame the combined effects of early sequestration and subsequent clearance enhancement, highlighting that fixed-dose escalation alone is insufficient. Given that despite treatment, the mortality in critically ill patients with invasive aspergillosis is high, this represents a critical therapeutic failure (42–44).

A paradigm shift toward time-aware monitoring is therefore essential. Early therapeutic drug monitoring at 24–48 h can detect the impact of extracorporeal circuit sequestration and suppressed clearance, while repeat assessment at days 3–5 identifies the subsequent clearance increase and guides dose escalation. This approach focuses on anticipating and responding to predictable changes in drug disposition rather than waiting for steady state.

Genotype-informed dosing further refines this strategy: poor and intermediate metabolizers require cautious early dosing to mitigate the risk of drug accumulation, whereas normal and rapid metabolizers may require more aggressive adjustment as clearance rises (45). Where available, Bayesian forecasting tools incorporating time-varying models could further support personalized dosing strategies (46).

In centers lacking capacity for intensive therapeutic drug monitoring, alternative antifungal agents may represent a more practical option. Isavuconazole demonstrates more predictable linear kinetics with fewer drug-drug interactions, while liposomal amphotericin B bypasses hepatic metabolism and may provide more consistent exposure in unstable clinical settings. However, this consideration needs to be balanced against the superior efficacy and lung tissue penetration associated with voriconazole (9).

### Study limitations

This single-center study conducted predominantly in patients receiving venovenous ECMO may not be generalizable to other ECMO populations or management strategies. While relatively large for a prospective pharmacokinetic study in the ECMO setting, the sample size may have limited statistical power to detect subtle covariate effects or specific drug interactions. In particular, the small number of poor metabolizers (*n* = 1) and ultrarapid metabolizers (*n* = 1) limited our ability to fully characterize the full spectrum of pharmacogenomic effects.

The dual-pathway model incorporating both sequestration and time-varying clearance provides an empirical description of drug disposition but cannot disentangle precise mechanistic contributions. Sequestration was parameterized as an apparent clearance term rather than measured directly by mass balance, and estimates for its magnitude and half-life were imprecise, limiting confidence in the specific quantitative contributions of circuit sequestration versus metabolic changes. Similarly, multiple biological processes—including inflammatory resolution, enzyme induction, and hemodynamic recovery—likely contributed to the observed clearance trajectory but could not be individually quantified. The mechanistic interpretation presented above therefore represents a plausible hypothesis consistent with known pathophysiology, rather than definitive causal attribution. The near-universal corticosteroid exposure in our cohort (87% of patients) prevented direct quantification of enzyme induction effects, and individual contributions from inflammation resolution, hemodynamic recovery, and steroid-mediated induction could not be separated in the current data set.

All patients received a proton pump inhibitor throughout the study period (intravenous omeprazole or enteral lansoprazole), precluding covariate analysis of CYP2C19 inhibition effects on clearance.

### Broader implications and future directions

The phenomenon of time-varying clearance likely extends beyond voriconazole to other lipophilic, hepatically metabolized drugs used in intensive practice (Fig. 4). Reports of similar patterns with midazolam and morphine during ECMO support the interpretation that dynamic pharmacokinetics reflects a general feature of critical illness and recovery rather than a drug-specific effect (47–49). This principle may inform dosing strategies for other drugs used in intensive care. A recent Delphi study conducted by the ECMO pharmacology network has identified appropriate antimicrobial dosing and pharmacokinetic/pharmacodynamic reporting as key research priorities aimed toward improving patient outcomes. The results of the present study would contribute to addressing these research priorities (50).

Future research priorities include multicenter validation across diverse ECMO populations and management protocols. Mechanistic studies incorporating serial inflammatory biomarkers, hepatic perfusion imaging, and cytochrome P450 activity probes could quantify individual contributions to clearance transitions. Development of predictive algorithms using routine clinical markers (C-reactive protein, bilirubin, lactate) could enable bedside anticipation of clearance changes without the need for sophisticated modeling software.

### Conclusion

Voriconazole clearance in patients receiving ECMO support is dynamic: early drug loss potentially attributable to circuit sequestration is followed by marked changes in intrinsic clearance, with CYP2C19 genotype showing clinically relevant associations with late-phase clearance. This temporal variability may explain previous conflicting reports and the frequent inadequacy of standard intravenous voriconazole dosing. Early and repeated therapeutic drug monitoring, coupled with proactive anticipation of clearance changes, is essential for achieving effective antifungal therapy in patients receiving ECMO. Acknowledging time-dependent pharmacokinetics may improve dosing precision in critical care, extending beyond voriconazole.

## SUPPLEMENTAL MATERIAL

Supplemental material aac.00098-26-s0001.docx Table S1; Fig. S1 to S5.

ASM does not own the copyrights to Supplemental Material that may be linked to, or accessed through, an article. The authors have granted ASM a non-exclusive, world-wide license to publish the Supplemental Material files. Please contact the corresponding author directly for reuse.

## References

[1] Gaffney S, Kelly DM, Rameli PM, Kelleher E, Martin-Loeches I. 2023. Invasive pulmonary aspergillosis in the intensive care unit: current challenges and best practices. APMIS 131:654–667. doi:10.1111/apm.13316 37022291

[2] Baddley JW, Stephens JM, Ji X, Gao X, Schlamm HT, Tarallo M. 2013. Aspergillosis in Intensive Care Unit (ICU) patients: epidemiology and economic outcomes. BMC Infect Dis 13:29. doi:10.1186/1471-2334-13-29 23343366 PMC3562254

[3] van de Veerdonk FL, Kolwijck E, Lestrade PPA, Hodiamont CJ, Rijnders BJA, van Paassen J, Haas P-J, Oliveira Dos Santos C, Kampinga GA, Bergmans D, van Dijk K, de Haan AFJ, van Dissel J, van der Hoeven HG, Verweij PE, Dutch Mycoses Study Group. 2017. Influenza-associated aspergillosis in critically ill patients. Am J Respir Crit Care Med 196:524–527. doi:10.1164/rccm.201612-2540LE 28387526

[4] Feys S, Carvalho A, Clancy CJ, Gangneux J-P, Hoenigl M, Lagrou K, Rijnders BJA, Seldeslachts L, Vanderbeke L, van de Veerdonk FL, Verweij PE, Wauters J. 2024. Influenza-associated and COVID-19-associated pulmonary aspergillosis in critically ill patients. Lancet Respir Med 12:728–742. doi:10.1016/S2213-2600(24)00151-6 39025089

[5] Wauters J, Baar I, Meersseman P, Meersseman W, Dams K, De Paep R, Lagrou K, Wilmer A, Jorens P, Hermans G. 2012. Invasive pulmonary aspergillosis is a frequent complication of critically ill H1N1 patients: a retrospective study. Intensive Care Med 38:1761–1768. doi:10.1007/s00134-012-2673-2 22895826 PMC7079899

[6] Schauwvlieghe A, Rijnders BJA, Philips N, Verwijs R, Vanderbeke L, Van Tienen C, Lagrou K, Verweij PE, Van de Veerdonk FL, Gommers D, Spronk P, Bergmans D, Hoedemaekers A, Andrinopoulou E-R, van den Berg C, Juffermans NP, Hodiamont CJ, Vonk AG, Depuydt P, Boelens J, Wauters J, Dutch-Belgian Mycosis study group. 2018. Invasive aspergillosis in patients admitted to the intensive care unit with severe influenza: a retrospective cohort study. Lancet Respir Med 6:782–792. doi:10.1016/S2213-2600(18)30274-1 30076119

[7] Alessandri F, Giordano G, Sanda VC, D’Ettorre G, Pugliese F, Ceccarelli G. 2025. Outcomes of severe aspergillosis in patients undergoing extracorporeal membrane oxygenation: a systematic review. Artif Organs 49:362–372. doi:10.1111/aor.14871 39310994

[8] Cavayas YA, Yusuff H, Porter R. 2018. Fungal infections in adult patients on extracorporeal life support. Crit Care 22:98. doi:10.1186/s13054-018-2023-z 29665838 PMC5905180

[9] Herbrecht R, Denning DW, Patterson TF, Bennett JE, Greene RE, Oestmann J-W, Kern WV, Marr KA, Ribaud P, Lortholary O, Sylvester R, Rubin RH, Wingard JR, Stark P, Durand C, Caillot D, Thiel E, Chandrasekar PH, Hodges MR, Schlamm HT, Troke PF, de Pauw B, Invasive Fungal Infections Group of the European Organisation for Research and Treatment of Cancer and the Global Aspergillus Study Group. 2002. Voriconazole versus amphotericin B for primary therapy of invasive aspergillosis. N Engl J Med 347:408–415. doi:10.1056/NEJMoa020191 12167683

[10] Ruiz J, Gordon M, Villarreal E, Peruccioni M, Marqués MR, Poveda-Andrés JL, Castellanos-Ortega Á, Ramirez P. 2019. Impact of voriconazole plasma concentrations on treatment response in critically ill patients. J Clin Pharm Ther 44:572–578. doi:10.1111/jcpt.12817 30851209

[11] Gatti M, Fornaro G, Pasquini Z, Zanoni A, Bartoletti M, Viale P, Pea F. 2023. Impact of inflammation on voriconazole exposure in critically ill patients affected by probable COVID-19-associated pulmonary aspergillosis. Antibiotics (Basel) 12:764. doi:10.3390/antibiotics12040764 37107125 PMC10134964

[12] Peek GJ, Mugford M, Tiruvoipati R, Wilson A, Allen E, Thalanany MM, Hibbert CL, Truesdale A, Clemens F, Cooper N, Firmin RK, Elbourne D, CESAR trial collaboration. 2009. Efficacy and economic assessment of conventional ventilatory support versus extracorporeal membrane oxygenation for severe adult respiratory failure (CESAR): a multicentre randomised controlled trial. Lancet 374:1351–1363. doi:10.1016/S0140-6736(09)61069-2 19762075

[13] Combes A, Hajage D, Capellier G, Demoule A, Lavoué S, Guervilly C, Da Silva D, Zafrani L, Tirot P, Veber B, et al.. 2018. Extracorporeal membrane oxygenation for severe acute respiratory distress syndrome. N Engl J Med 378:1965–1975. doi:10.1056/NEJMoa1800385 29791822

[14] Raffaeli G, Cavallaro G, Allegaert K, Koch BCP, Mosca F, Tibboel D, Wildschut ED. 2020. Sequestration of voriconazole and vancomycin into contemporary extracorporeal membrane oxygenation circuits: an in vitro study. Front Pediatr 8:468. doi:10.3389/fped.2020.00468 32974242 PMC7481439

[15] Lyster H, Pitt T, Maunz O, Diamond S, Roberts JA, Brown D, Mills J, Armstrong-James D, Gerovasili V, Carby M, Dunning J, Simon A, Reed A. 2023. Variable sequestration of antifungals in an extracorporeal membrane oxygenation circuit. ASAIO J 69:309–314. doi:10.1097/MAT.0000000000001802 36731055

[16] Zhang Y, Hu H, Zhang Q, Ou Q, Zhou H, Sha T, Zeng Z, Wu J, Lu J, Chen Z. 2021. Effects of ex vivo extracorporeal membrane oxygenation circuits on sequestration of antimicrobial agents. Front Med 8. doi:10.3389/fmed.2021.748769 PMC8671752 34926498

[17] Mathieu A, Thiboutot Z, Ferreira V, Benoit P, Grandjean Lapierre S, HÉtu P-O, Halwagi A. 2022. Voriconazole sequestration during extracorporeal membrane oxygenation for invasive lung aspergillosis: a case report. ASAIO J 68:e56–e58. doi:10.1097/MAT.0000000000001427 33788798

[18] Gautier-Veyret E, Truffot A, Bailly S, Fonrose X, Thiebaut-Bertrand A, Tonini J, Cahn J-Y, Stanke-Labesque F. 2019. Inflammation is a potential risk factor of voriconazole overdose in hematological patients. Fundam Clin Pharmacol 33:232–238. doi:10.1111/fcp.12422 30306637

[19] Peterson EL, Chittick PJ, Richardson CL. 2021. Decreasing voriconazole requirement in a patient after extracorporeal membrane oxygenation discontinuation: a case report. Transpl Infect Dis 23:e13545. doi:10.1111/tid.13545 33316840

[20] Spriet I, Annaert P, Meersseman P, Hermans G, Meersseman W, Verbesselt R, Willems L. 2009. Pharmacokinetics of caspofungin and voriconazole in critically ill patients during extracorporeal membrane oxygenation. J Antimicrob Chemother 63:767–770. doi:10.1093/jac/dkp026 19218271

[21] Brüggemann RJM, Antonius T, Heijst A van, Hoogerbrugge PM, Burger DM, Warris A. 2008. Therapeutic drug monitoring of voriconazole in a child with invasive aspergillosis requiring extracorporeal membrane oxygenation. Ther Drug Monit 30:643–646. doi:10.1097/FTD.0b013e3181898b0c 19057370

[22] Lin X-B, Hu X-G, Xia Y-Z, Liu X-M, Liang T, Chen X, Cai C-J. 2022. Voriconazole pharmacokinetics in a critically ill patient during extracorporeal membrane oxygenation. J Chemother 34:272–276. doi:10.1080/1120009X.2021.2014725 34904531

[23] Ye Q, Yu X, Chen W, Li M, Gu S, Huang L, Zhan Q, Wang C. 2022. Impact of extracorporeal membrane oxygenation on voriconazole plasma concentrations: a retrospective study. Front Pharmacol 13:972585. doi:10.3389/fphar.2022.972585 36059951 PMC9428491

[24] Van Daele R, Bekkers B, Lindfors M, Broman LM, Schauwvlieghe A, Rijnders B, Hunfeld NGM, Juffermans NP, Taccone FS, Coimbra Sousa CA, Jacquet L-M, Laterre P-F, Nulens E, Grootaert V, Lyster H, Reed A, Patel B, Meersseman P, Debaveye Y, Wauters J, Vandenbriele C, Spriet I. 2021. A large retrospective assessment of voriconazole exposure in patients treated with extracorporeal membrane oxygenation. Microorganisms 9:1543. doi:10.3390/microorganisms9071543 34361978 PMC8303158

[25] Lamoureux F, Duflot T, Woillard J-B, Metsu D, Pereira T, Compagnon P, Morisse-Pradier H, El Kholy M, Thiberville L, Stojanova J, Thuillez C. 2016. Impact of CYP2C19 genetic polymorphisms on voriconazole dosing and exposure in adult patients with invasive fungal infections. Int J Antimicrob Agents 47:124–131. doi:10.1016/j.ijantimicag.2015.12.003 26775563

[26] Moriyama B, Obeng AO, Barbarino J, Penzak SR, Henning SA, Scott SA, Agúndez JAG, Wingard JR, McLeod HL, Klein TE, Cross SJ, Caudle KE, Walsh TJ. 2017. Clinical pharmacogenetics implementation consortium (CPIC) guidelines for CYP2C19 and voriconazole therapy. Clin Pharmacol Ther 102:45–51. doi:10.1002/cpt.583 27981572 PMC5474211

[27] Shah RR, Smith RL. 2015. Inflammation-induced phenoconversion of polymorphic drug metabolizing enzymes: hypothesis with implications for personalized medicine. Drug Metab Dispos 43:400–410. doi:10.1124/dmd.114.061093 25519488

[28] Wallace KL, Filipek RL, La Hoz RM, Williamson JC. 2016. Subtherapeutic voriconazole concentrations associated with concomitant dexamethasone: case report and review of the literature. J Clin Pharm Ther 41:441–443. doi:10.1111/jcpt.12401 27207573

[29] Jia S-J, Gao K-Q, Huang P-H, Guo R, Zuo X-C, Xia Q, Hu S-Y, Yu Z, Xie Y-L. 2021. Interactive effects of glucocorticoids and cytochrome P450 polymorphisms on the plasma trough concentrations of voriconazole. Front Pharmacol 12:666296. doi:10.3389/fphar.2021.666296 34113252 PMC8185288

[30] Huang J, Chen Y, Zhong M, Tan R. 2024. Case report: dose-dependent interaction between dexamethasone and voriconazole in severely ill patients with non-Hodgkin’s lymphoma being treated for invasive pulmonary aspergillosis. Front Pharmacol 15:1403966. doi:10.3389/fphar.2024.1403966 38994198 PMC11236688

[31] Le Daré B, Boglione-Kerrien C, Reizine F, Gangneux JP, Bacle A. 2021. Toward the personalized and integrative management of voriconazole dosing during COVID-19-associated pulmonary aspergillosis. Crit Care 25:152. doi:10.1186/s13054-021-03568-8 33879175 PMC8056831

[32] Lenoir C, Rollason V, Desmeules JA, Samer CF. 2021. Influence of inflammation on cytochromes P450 activity in adults: a systematic review of the literature. Front Pharmacol 12:733935. doi:10.3389/fphar.2021.733935 34867341 PMC8637893

[33] Takesue Y, Hanai Y, Oda K, Hamada Y, Ueda T, Mayumi T, Matsumoto K, Fujii S, Takahashi Y, Miyazaki Y, Kimura T, Japanese Antimicrobial Therapeutic Drug Monitoring Guideline Committee. 2022. Clinical practice guideline for the therapeutic drug monitoring of voriconazole in non-Asian and Asian adult patients: consensus review by the Japanese Society of Chemotherapy and the Japanese Society of Therapeutic Drug Monitoring. Clin Ther 44:1604–1623. doi:10.1016/j.clinthera.2022.10.005 36424314

[34] Ullmann AJ, Aguado JM, Arikan-Akdagli S, Denning DW, Groll AH, Lagrou K, Lass-Flörl C, Lewis RE, Munoz P, Verweij PE, et al.. 2018. Diagnosis and management of Aspergillus diseases: executive summary of the 2017 ESCMID-ECMM-ERS guideline. Clin Microbiol Infect 24:e1–e38. doi:10.1016/j.cmi.2018.01.002 29544767

[35] Wichmann D, Hoenigl M, Koehler P, Koenig C, Lund F, Mang S, Strauß R, Weigand MA, Hohmann C, Kurzai O, Heußel C, Kochanek M. 2025. Diagnosis and treatment of invasive pulmonary aspergillosis in critically ill intensive care patients: executive summary of the German national guideline (AWMF 113-005). Infection 53:1299–1310. doi:10.1007/s15010-025-02572-2 40465080 PMC12316785

[36] Koehler P, Bassetti M, Chakrabarti A, Chen SCA, Colombo AL, Hoenigl M, Klimko N, Lass-Flörl C, Oladele RO, Vinh DC, et al.. 2021. Defining and managing COVID-19-associated pulmonary aspergillosis: the 2020 ECMM/ISHAM consensus criteria for research and clinical guidance. Lancet Infect Dis 21:e149–e162. doi:10.1016/S1473-3099(20)30847-1 33333012 PMC7833078

[37] Yusuff H, University Hospitals of Leicester NHS Trust. 2004. Guidelines for use of anti-fungal agents on adult patients with vital pneumonitis requiring Extracorporeal Membrane Oxygenation (ECMO)

[38] Wang Y, Ye Q, Li P, Huang L, Qi Z, Chen W, Zhan Q, Wang C. 2014. Renal replacement therapy as a new indicator of voriconazole clearance in a population pharmacokinetic analysis of critically ill patients. Pharmaceuticals (Basel) 17:665. doi:10.3390/ph17060665 PMC11206427 38931333

[39] Shekar K, Abdul-Aziz MH, Cheng V, Burrows F, Buscher H, Cho Y-J, Corley A, Diehl A, Gilder E, Jakob SM, Kim H-S, Levkovich BJ, Lim SY, McGuinness S, Parke R, Pellegrino V, Que Y-A, Reynolds C, Rudham S, Wallis SC, Welch SA, Zacharias D, Fraser JF, Roberts JA. 2023. Antimicrobial exposures in critically ill patients receiving extracorporeal membrane oxygenation. Am J Respir Crit Care Med 207:704–720. doi:10.1164/rccm.202207-1393OC 36215036

[40] Klomp SD, Veringa A, Alffenaar J-W, de Boer MGJ, Span LFR, Guchelaar H-J, Swen JJ. 2024. Inflammation altered correlation between CYP2C19 genotype and CYP2C19 activity in patients receiving voriconazole. Clin Transl Sci 17:e13887. doi:10.1111/cts.13887 39010708 PMC11250525

[41] Shekar K, Fraser JF, Smith MT, Roberts JA. 2012. Pharmacokinetic changes in patients receiving extracorporeal membrane oxygenation. J Crit Care 27:741. doi:10.1016/j.jcrc.2012.02.013 22520488

[42] Paiva JA, Mergulhão P, Gomes A, Taccone FS, Van den Abeele A-M, Bulpa P, Misset B, Meersseman W, Dimopoulos G, Rello J, Vogelaers D, Blot S. 2017. Drivers and impact of antifungal therapy in critically ill patients with Aspergillus-positive respiratory tract cultures. Int J Antimicrob Agents 50:529–535. doi:10.1016/j.ijantimicag.2017.05.017 28669830

[43] Barchiesi F, Santinelli A, Biscotti T, Greganti G, Giannini D, Manso E. 2016. Delay of antifungal therapy influences the outcome of invasive aspergillosis in experimental models of infection. J Antimicrob Chemother 71:2230–2233. doi:10.1093/jac/dkw111 27231274

[44] Delsuc C, Cottereau A, Frealle E, Bienvenu A-L, Dessein R, Jarraud S, Dumitrescu O, Le Maréchal M, Wallet F, Friggeri A, Argaud L, Rimmelé T, Nseir S, Ader F. 2015. Putative invasive pulmonary aspergillosis in critically ill patients with chronic obstructive pulmonary disease: a matched cohort study. Crit Care 19:421. doi:10.1186/s13054-015-1140-1 26631029 PMC4668635

[45] Hamadeh IS, Klinker KP, Borgert SJ, Richards AI, Li W, Mangal N, Hiemenz JW, Schmidt S, Langaee TY, Peloquin CA, Johnson JA, Cavallari LH. 2017. Impact of the CYP2C19 genotype on voriconazole exposure in adults with invasive fungal infections. Pharmacogenet Genomics 27:190–196. doi:10.1097/FPC.0000000000000277 28306618 PMC5391994

[46] Abrantes JA, Jönsson S, Karlsson MO, Nielsen EI. 2019. Handling interoccasion variability in model-based dose individualization using therapeutic drug monitoring data. Br J Clin Pharmacol 85:1326–1336. doi:10.1111/bcp.13901 30767254 PMC6533430

[47] Peters JWB, Anderson BJ, Simons SHP, Uges DRA, Tibboel D. 2005. Morphine pharmacokinetics during venoarterial extracorporeal membrane oxygenation in neonates. Intensive Care Med 31:257–263. doi:10.1007/s00134-004-2545-5 15678314

[48] Ahsman MJ, Hanekamp M, Wildschut ED, Tibboel D, Mathot RAA. 2010. Population pharmacokinetics of midazolam and its metabolites during venoarterial extracorporeal membrane oxygenation in neonates. Clin Pharmacokinet 49:407–419. doi:10.2165/11319970-000000000-00000 20481651

[49] Mulla H, Lawson G, Peek GJ, Firmin RK, Upton DR. 2003. Plasma concentrations of midazolam in neonates receiving extracorporeal membrane oxygenation. ASAIO J 49:41–47. doi:10.1097/00002480-200301000-00007 12558306

[50] Morales Castro D, Al-Fares AA, Paternoster G, Lyster H, Hohlfelder B, Arias Ortiz J, Abdul-Aziz MH, Herr D, Alraish R, Ferre Contreras A, et al.. 2025. Pharmacological research agenda on adult extracorporeal membrane oxygenation using the Delphi method: a position paper of the extracorporeal membrane oxygenation pharmacology network. Crit Care Med 53:e2246–e2260. doi:10.1097/CCM.0000000000006806 40736325

[51] legislation.gov.uk. 2005. Mental capacity act. https://www.legislation.gov.uk/ukpga/2005/9/contents.

