# Supplementary material for: Time-varying voriconazole clearance during extracorporeal membrane oxygenation
Source: Antimicrob Agents Chemother. 2026 Apr 30;70(6):e00098-26. doi: 10.1128/aac.00098-26 (PMC13231916; doi:10.1128/aac.00098-26)
Supplement: Supplemental material — Table S1; Fig. S1 to S5. [file aac.00098-26-s0001.docx]

# Supplementary Tables

## Table S1. Laboratory Parameters

| Variable | Mean | Median | Range |
| --- | --- | --- | --- |
| Voriconazole, mg/L | 3.14 | 3.2 | 0.1–8.0 |
| Sodium, mmol/L | 140.6 | 140 | 131–155 |
| Potassium, mmol/L | 4.3 | 4.3 | 3.2–5.2 |
| Urea, mmol/L | 10.1 | 10.2 | 1.9–24 |
| Creatinine, µmol/L | 93.2 | 84 | 18–273 |
| Bilirubin, µmol/L | 27.3 | 15 | 3–128 |
| Albumin, g/L | 27.1 | 27 | 16–41 |
| GGT, U/L | 150.1 | 108 | 10–646 |
| ALT, U/L | 83.2 | 63 | 8–526 |
| ALP, U/L | 133.1 | 106.5 | 43–358 |
| Haematocrit | 0.30 | 0.30 | 0.20–0.37 |
| CRP, mg/L | 137.1 | 105 | 5–409 |
| Procalcitonin, µg/L | 25.0 | 0.8 | 0.02–480 |

Table S1. Laboratory parameters. Values are mean, median, and range as observed during study participation.

**Monolix Model code**

DESCRIPTION:

One-compartment voriconazole population pharmacokinetic model for adults receiving ECMO. Total clearance comprises two parallel time-varying pathways: intrinsic clearance rising logistically from CL1 to CL2, and an exponentially decaying sequestration clearance (CLseq0). Volume of distribution is scaled by body weight.

[LONGITUDINAL]

input = {

V, ; central volume

CL1, CL2, ; intrinsic clearance: early level (CL1) and late level (CL2)

Tswitch, ; logistic midpoint (h)

CLseq0, ; sequestration-related clearance at t=0

T12seq, ; sequestration half-life (h)

WT

}

WT = {use=regressor}

EQUATION:

odeType = stiff

V_WT = V * (WT/70)

; --- Intrinsic clearance: logistic from CL1 -> CL2 ---

sigmoid = 1/(1 + exp(-0.1*(t - Tswitch))) ; 0.1 = steepness (1/h)

CL_intr = CL1 + sigmoid * (CL2 - CL1)

; --- Sequestration "apparent" clearance: high early, decays mono-exponentially ---

k_seq = log(2)/T12seq

CL_seq = CLseq0 * exp(-k_seq * t)

; --- Total clearance (parallel pathways) ---

CL_time = CL_intr + CL_seq

Cc = pkmodel(V=V_WT, Cl=CL_time)

OUTPUT:

output = {Cc}


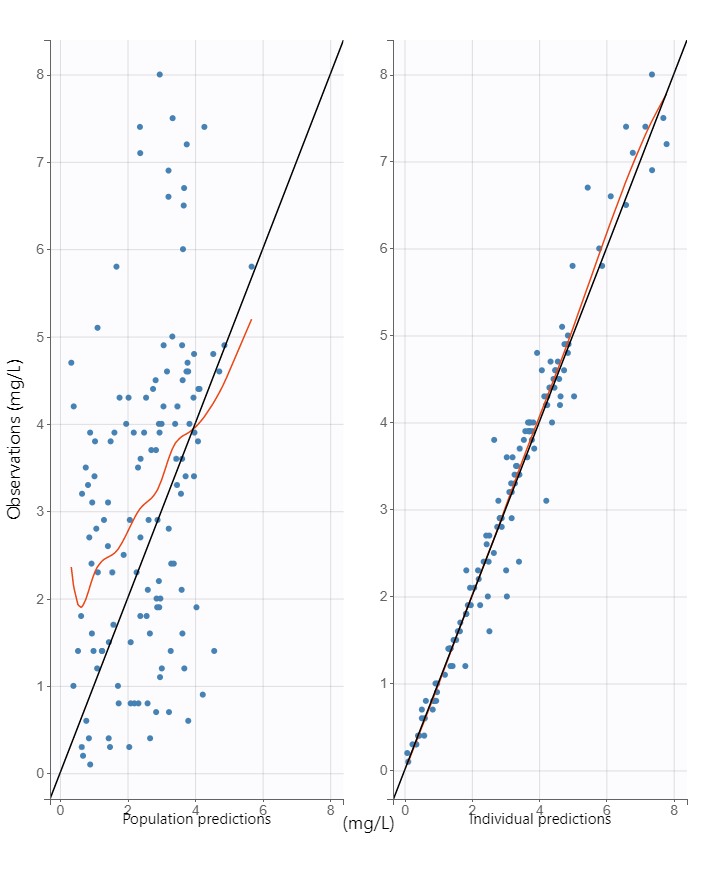


Figure S1. Observed versus model-predicted voriconazole concentrations based on population (left) and individual (right) predictions and the red spline indicates locally weighted smooth trend. The closer alignment of observations with the line of identity in the individual prediction plot indicates that the final model provides a good individual fit and captures interindividual variability accurately.


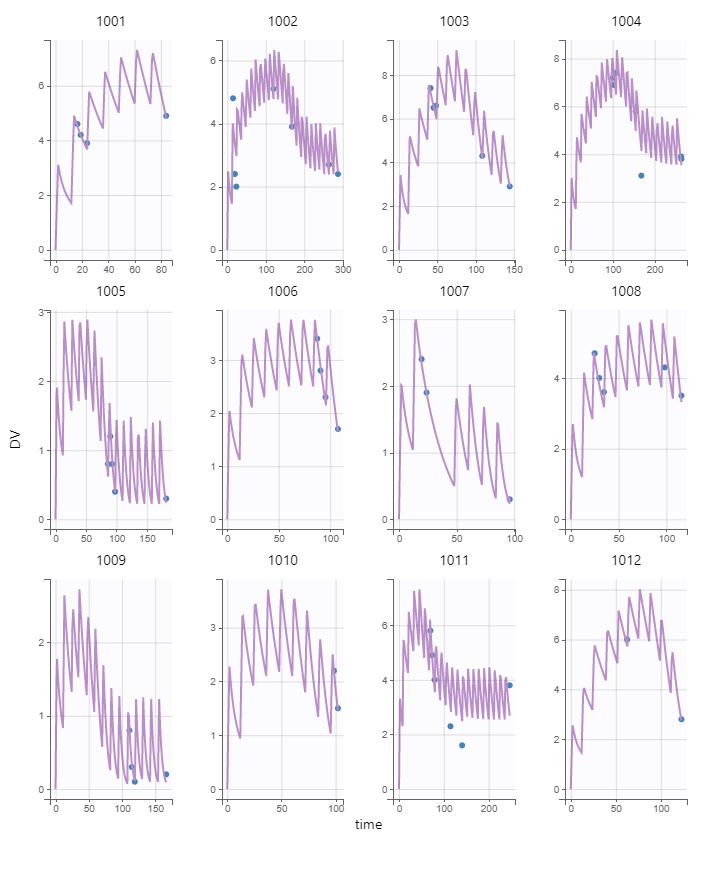

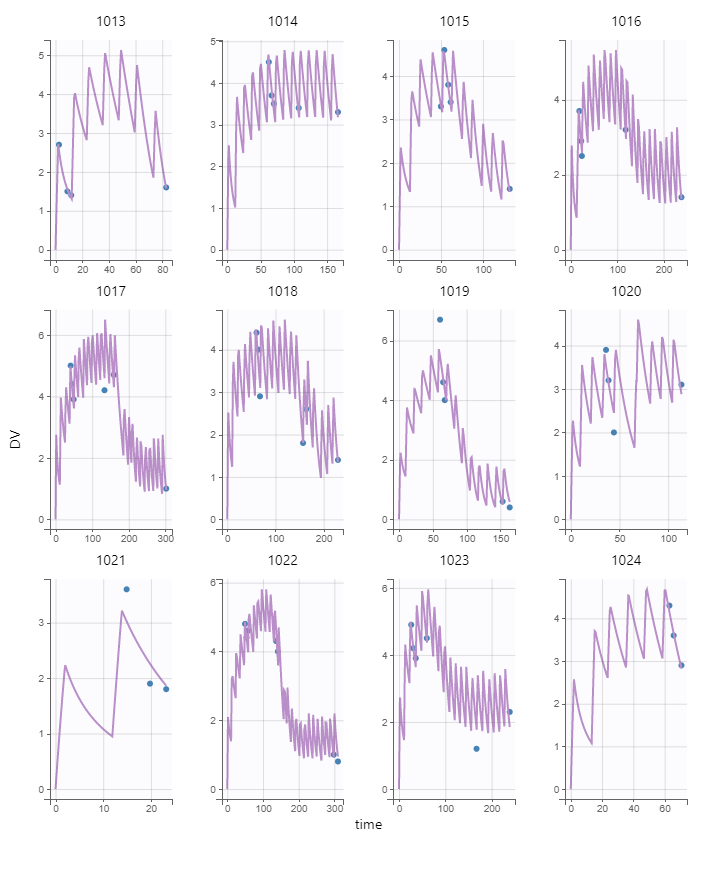

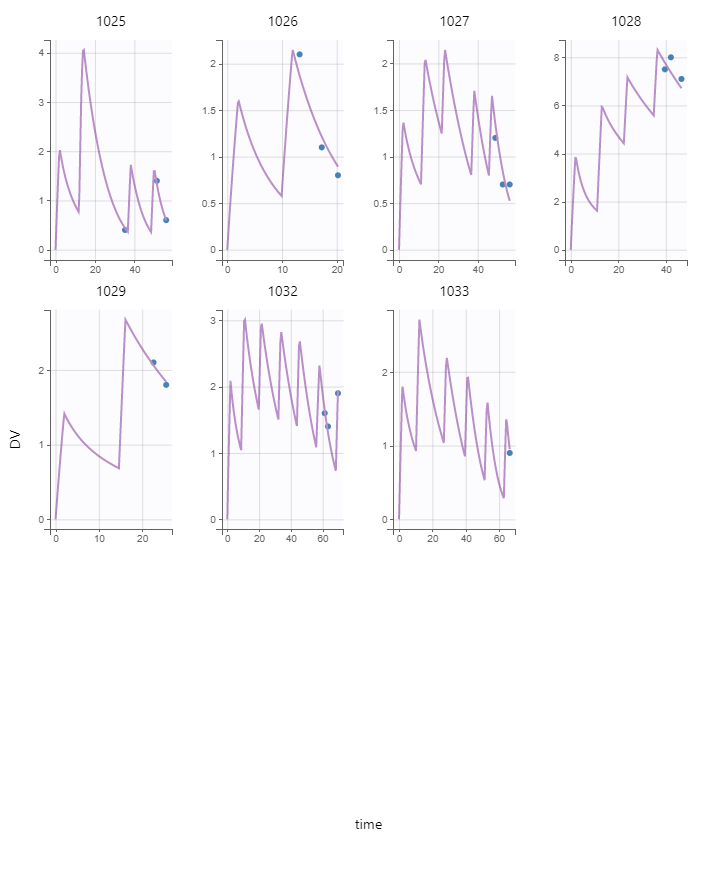


Figure S2. Individual observed (points) and model-predicted (lines) voriconazole concentration–time profiles for representative patients receiving ECMO. The final dual-pathway model (incorporating circuit sequestration and time-varying clearance) adequately captured both early-phase concentration decline and subsequent variability across individuals.


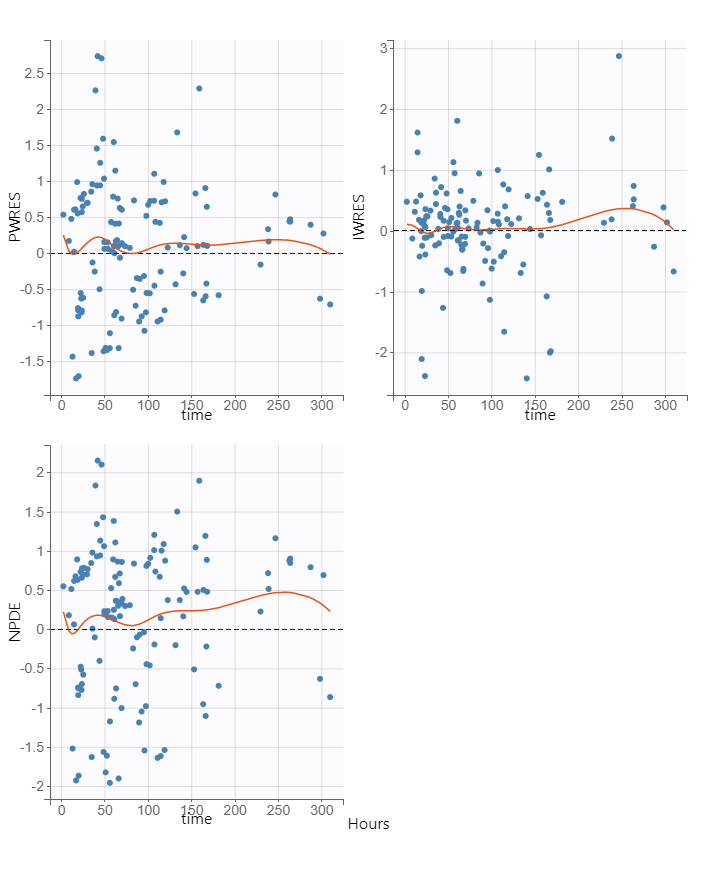


Figure S3. Diagnostic residual plots for the final population pharmacokinetic model of voriconazole during ECMO. Population weighted residuals (PWRES), individual weighted residuals (IWRES), and normalised prediction distribution errors (NPDE) are plotted against time. The red spline lines represent locally weighted smooth trends. Residuals were symmetrically distributed around zero with no systematic bias over time or concentration range, confirming model adequacy.


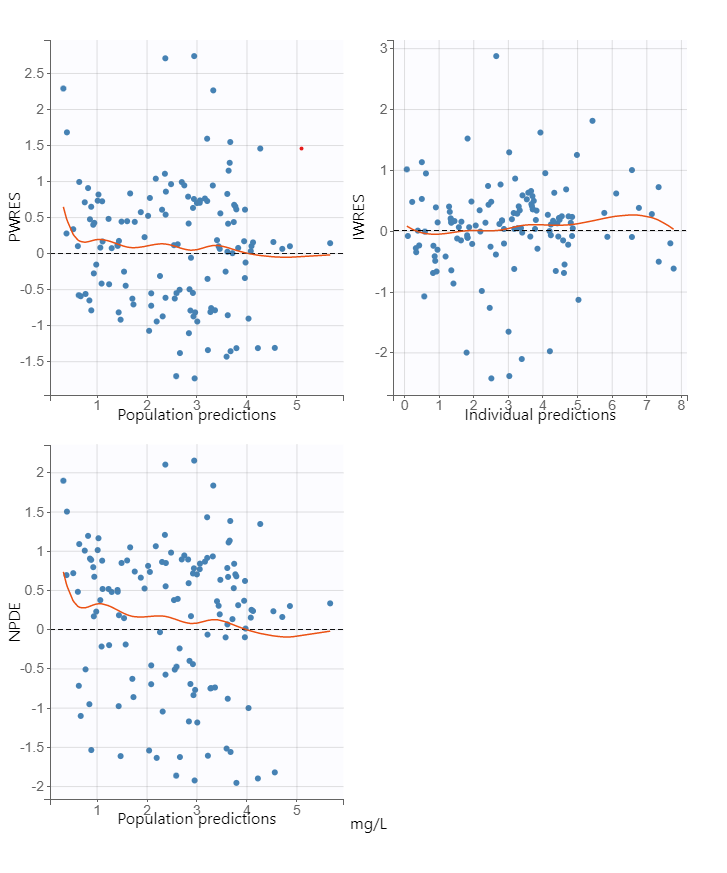


Figure S4. Diagnostic residual plots for the final population pharmacokinetic model of voriconazole during ECMO. Population weighted residuals (PWRES), individual weighted residuals (IWRES), and normalised prediction distribution errors (NPDE) are plotted against predictions. The red spline lines represent locally weighted smooth trends. Residuals were symmetrically distributed around zero with no systematic bias over time or concentration range, confirming model adequacy.


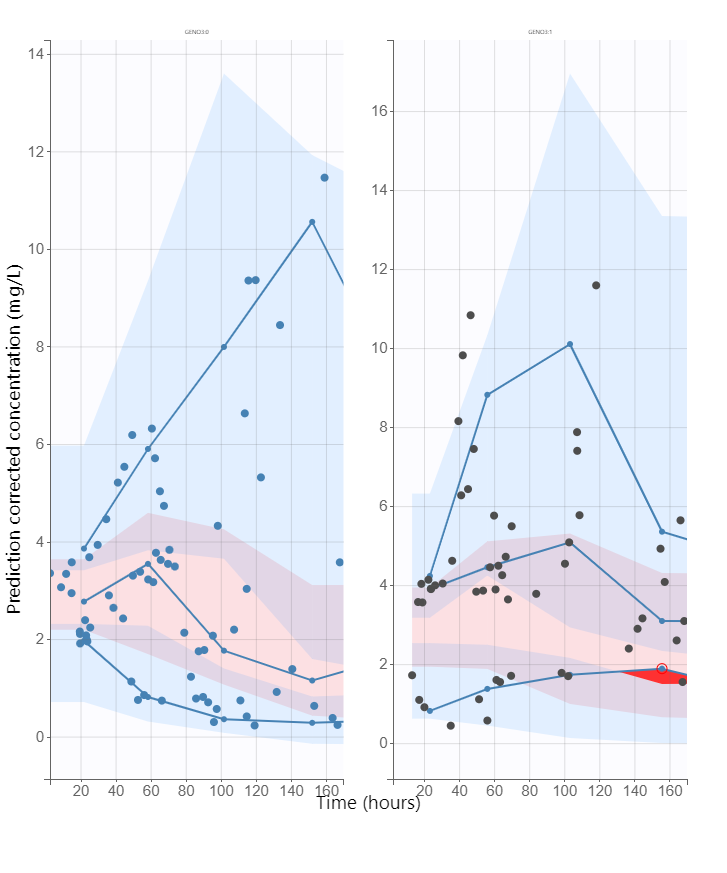
Figure S5. Prediction-corrected visual predictive checks for voriconazole stratified by CYP2C19 genotype. Observed prediction-corrected concentrations (points) and empirical 5th, 50th, and 95th percentiles (solid blue lines) are shown for wild-type (left, blue points) and intermediate/poor metaboliser groups (right, black points). Shaded areas represent the 90% prediction intervals for simulated data (blue for 5th and 95th percentiles, pink for the median). The model reproduced the central tendency and variability well in the wild-type group, while predictive precision was lower in the intermediate/poor metaboliser group due to fewer observations.
